# A draft genome for a species of *Halicephalobus* (Panagrolaimidae)

**DOI:** 10.21307/jofnem-2019-068

**Published:** 2019-10-14

**Authors:** Erik J. Ragsdale, Georgios Koutsovoulos, Joseph F. Biddle

**Affiliations:** 1 Department of Biology, Indiana University, 915 E. 3rd St., Bloomington, IN, 47405; 2 Institute national de la recherche agronomique, Sophia-Antipolis, PACA, France

**Keywords:** Comparative genomics, Free-living, Panagrolaimorpha, Parthenogenesis

## Abstract

*Halicephalobus* is a clade of small, exclusively parthenogenic nematodes that have sometimes colonized remarkable habitats. Given their phylogenetic closeness to other parthenogenic panagrolaimid species with which they likely share a sexually reproducing ancestor, *Halicephalobus* species provide a point of comparison for parallelisms in the evolution of asexuality. Here, we present a draft genome of a putatively new species of *Halicephalobus* isolated from termites in Japan.

Panagrolaimidae are a nematode family represented by lifestyles under the most extreme environments known to animals, including desiccation (Ricci and Pagani 1997; Treonis and Wall, 2005), freezing (Wharton and Barclay, 1993), and an unusual range of pH conditions (Peters, 1928). Phylogenetically close families that include important parasites of insects and vertebrates, Steinernematidae and Strongyloididae, respectively, have enjoyed considerable attention from sequencing efforts. However, free-living species of Panagrolaimidae have lagged behind, as have free-living species of Clade IV (*sensu* Blaxter et al., 1998) more generally. A major contribution to the genomics of Panagrolaimidae was the genome sequence of *Panagrellus redivivus*, a species championed for decades as a laboratory model for genetics and development, especially as a satellite to *Caenorhabditis elegans* (Srinivasan et al., 2013). More recently, several species of *Panagrolaimus* have been sequenced to study the evolutionary signatures of parthenogenesis and tolerance of extreme environments (Schiffer et al. under review). To provide resources for another member of this nematode family, we present a draft genome for a strain of *Halicephalobus*.

*Halicephalobus* species are minute nematodes that often inhabit soil-like environments and are particularly well-known from organic-rich substrates (Andrássy, 1984; Anderson et al., 1998; Steel et al., 2012). Also, like other panagrolaimids, these nematodes have been reported from demanding, unusual habitats. For example, *H. gingivalis*, typically a resident of soil and humus, is an opportunistic, blood-inhabiting pathogen of mammals, especially horses and occasionally humans (Anderson and Bemrick, 1965; Hoogstraten and Young, 1975; Blunden et al., 1987). Another species of this genus, *H. mephisto*, was discovered on microbial biofilms at depths of over three kilometers within the earth’s crust (Borgonie et al., 2011). The ability of *Halicephalobus* species to colonize such habitats suggests that comparative genomics of the genus may yield clues into the mechanisms enabling unusual lifestyles. It is possible that the mode of reproduction in all known *Halicephalobus* species, obligate parthenogenesis, also aids in the colonization of new, sometimes extreme habitats, by requiring only a single individual to found a population. Because the common ancestor of *Panagrolaimus* and *Halicephalobus* was most likely gonochoristic (dioecious) (Lewis et al., 2009), parthenogenesis may have evolved twice independently in these two genera. Consequently, the genome for a *Halicephalobus* species may reveal illuminating parallelisms in how asexuality influences genomic evolution.

Here, we have sequenced the genome of a *Halicephalobus* strain (NKZ332) isolated from termites in Japan. Because of the paucity of reliable diagnostic morphology for the genus (Anderson et al., 1998), combined with the inability to test species boundaries through crossing experiments, assignment of a valid species binomial to this or other species of the genus is not infallible. Indeed, taxonomic revision of this genus will rely on molecular data (Nadler et al., 2003), interpreted in terms of evolutionary or phylogenetic species concepts (e.g., Borgonie et al., 2011). Based on bionomic evidence, specifically the nematodes’ colonization of rotting wood, the sequenced isolate herein would be best assigned to *H. similigaster*, a morphospecies known from Europe (Andrássy, 1952; Köhler, 2011). However, the present strain shows a sharp distinction in rRNA markers, specifically <95% similarity, from all other sequenced *Halicephalobus* isolates, particularly those from similar habitats in other locations (Foley et al., 2018). This similarity is much less than that between biological species in other nematode groups (Kanzaki et al., 2013; Félix et al., 2014). Given its molecular and geographic distinctness from other known *Halicephalobus* strains and morphospecies, the present strain likely has an evolutionary fate (*sensu* Wiley, 1978) separate from other nominal species. Therefore, we anticipate that molecular comparisons of *Halicephalobus* strains from both Europe and East Asia will support and delimit *Halicephalobus* sp. NKZ332 as a new species.

To sequence the genome of *Halicephalobus* sp. NKZ332, which we collected from xenic bacterial culture, we used the Illumina NextSeq platform to generate 75 million, 300-bp, paired-end reads of 150-bp and 500-bp insert sizes. Reads were trimmed with Cutadapt (Martin, 2011) and error-corrected with Reckoner (Długosz and Deorowicz, 2017). We first used Minia (Drezen et al., 2014) to perform a preliminary single-end assembly, with which we validated insert size and screened for possible non-nematode contaminants using Blobtools (Kumar et al., 2013). We identified two contaminants, *Stenotophomonas maltophilia* at high coverage and *Pseudomonas sp.* at low coverage, both with a higher GC content than the predicted nematode scaffolds’ GC content (38%). We then assembled the genome using the SPAdes assembler (Bankevich et al., 2012) and removed scaffolds that were identified as contamination, at low coverage (<10×), or less than 500 bp in length. We made a first-pass annotation with the MAKER pipeline using evidence drawn from the Swiss-Prot database, the hidden Markov model (HMM) profile computed from GeneMark (Lukashin and Borodovsky, 1998), and the Augustus (Stanke and Waack, 2003) profile generated with BUSCO (Waterhouse et al., 2018). We then used the predicted genes from the MAKER output to train and run Augustus to produce the final annotation.

Our assembly of *Halicephalobus* sp. NKZ332 spans 47 Mb in 5,085 contigs with an N50 of 60 kb. This assembly size is smaller than the genomes of most sequenced nematodes (International Helminth Genomes Consortium, 2018). Likewise, the assembly is much smaller than those of other *Panagrolaimus* species, whose genomes are ~140 to ~180 Mb in gonochoristic species and even larger (~230–270 Mb) in parthenogenic strains, which likely originated from an allopolyploidy event (Schiffer et al. under review). In our annotation, we predicted a total of 11,023 genes with a mean of 1,511 bp in length. We assessed the completeness of the genome assembly using BUSCO (Simão et al. 2015), with which our annotation shared 83.2% (79.8% complete genes) of the nematode ortholog set. Given such representation of conserved orthologs in the annotation, the genome of *Halicephalobus* sp. NKZ332 is strikingly minimal. Finally, using the MITOS Web Server (Bernt et al. 2013), we assembled the mitochondrial genome sequence, which has a length of 13,886 bp and GC content of 20%.

In summary, we present a compact nematode genome representing a genus of small, parthenogenic, and ecologically opportunistic nematodes. The nuclear and mitochondrial genome sequences have been deposited in NCBI GenBank under accession numbers VOSG00000000 and MN207311, respectively. Raw reads have been deposited in the NCBI Sequence Read Archive (SAMN12324525).
